# Developing an Ostrich Welfare Assessment Protocol (OWAP) in Intensive and Semi-Intensive Systems

**DOI:** 10.3390/vetsci12040380

**Published:** 2025-04-18

**Authors:** Annalisa Previti, Diego Antonio Sicuso, Vito Biondi, Abrha Bsrat, Michela Pugliese, Behiru Gebrekidan, Annamaria Passantino

**Affiliations:** 1 Department of Veterinary Sciences, University of Messina, 98168 Messina, Italy; annalisa.previti@yahoo.it (A.P.); diego150899@gmail.com (D.A.S.); vito.biondi@unime.it (V.B.); annamaria.passantino@unime.it (A.P.); 2 College of Veterinary Medicine, Mekelle University, Mekelle 231, Tigray, Ethiopia; abrha.bsrat@mu.edu.et (A.B.); behiru.gebrekidan@mu.edu.et (B.G.)

**Keywords:** ostriches, animal welfare, farming, protocol, parameters

## Abstract

In recent years, consumer demand for animal-friendly production systems has driven the livestock industry to improve welfare standards. Protocols have been developed for different species such as calves, pigs, laying hens, and broilers to assess farms, compare practices, and establish a baseline for optimal welfare. However, despite their undoubted importance and presence on farms, ostriches are not included in these protocols. This study aims to develop a protocol for the welfare assessment of ostriches reared in intensive and semi-intensive systems using different indicators, which is feasible and time efficient. The protocol developed combines animal-based measures (physiological, appearance, and behavioral) with resource-based and management-based measures (management and environmental). Specifically, the ostrich welfare assessment protocol (OWAP) included 41 non-invasive measures: 14 animal-based, 12 resource-based, and 15 management-based. These measures were selected for their relevance, reliability, and feasibility. Each parameter is scored on a graded scale based on its condition, and the scores are aggregated to determine the overall welfare status of the ostriches. A threshold is set to classify welfare as acceptable, suboptimal, or unacceptable. Out of a total score of 95 (64 for indirect measures and 31 for direct measures), a score <32 was considered unacceptable, between 32–63 suboptimal, and >63 optimal. This scoring system provides a systematic, objective approach to assessing ostrich welfare under different husbandry conditions.

## Simple Summary

Growing consumer demand for ethical, animal-friendly practices has driven the livestock industry to improve welfare standards. While protocols exist for species like calves, pigs, and poultry, ostriches are often excluded. This study develops a protocol to assess ostrich welfare in intensive and semi-intensive systems, using animal-based (physiological, appearance, and behavioral), resource-based, and management-based indicators. The protocol includes 41 non-invasive measures: 1 animal-based, 12 resource-based, and 15 management-based, selected for their relevance and feasibility. Each measure is scored on a graded scale, with the overall score determining whether the welfare is acceptable, suboptimal, or unacceptable.

## 1. Introduction

Animal welfare is a growing concern in modern agriculture, with increasing attention given to the well-being of farmed species, from the most conventional such as poultry and cattle to the newly introduced ones, such as ostriches.

This species belongs to the subclass of ratites and is the largest flightless bird in the world [1,2]. It has been domesticated more recently than other species and, therefore, represents a still-growing sector [2]. Historically, the main products obtained from these animals were feathers, which were mostly used for decorative purposes [3]; however, after the Second World War, a shift occurred, with the focus moving to products such as meat and leather [4]. The first one is highly sought after for its advantageous nutritional composition (high in protein and low in fat), and the second for its high resistance and quality [5,6]. In breeding, the most critical phase is the reproductive phase [7], but, in general, the management of these animals—in particular their nutrition, housing, and welfare—is of fundamental importance, as it significantly impacts both productivity and performance. Furthermore, it is important to remember that only proper management can prevent diseases and infections that could potentially affect the health of entire herds [8]. This favors the outbreak of diseases, often zoonotic and food-borne, which undoubtedly pose a risk to consumer health [9]. For this reason, animal welfare is also a key issue in ostrich farming, given the human consumption of its products (meat and eggs).

Various protocols have been developed to assess welfare conditions, each with unique features and methodologies. For example, the ClassyFarm system, introduced by the Italian Ministry of Health, is a comprehensive tool used to evaluate animal welfare on farms. This system highlights a significant relationship between high animal welfare standards and improved productive performance, such as final weight and average daily gain [10]. The Animal Welfare and Biosecurity Evaluation form (AWB-EF) is another checklist developed by the Italian National Centre of Reference for Animal Welfare. This checklist is considered a gold standard and highlights the importance of combining welfare assessments with laboratory parameters to detect stress conditions early [11]. The Italian National Animal Welfare Reference Center (CReNBA) checklist provides a numerical welfare index and has been used to study the correlation between welfare conditions and the prevalence of infectious diseases. The findings suggest that better management and housing conditions can reduce the prevalence of infections, thereby improving overall welfare [12].

In Austria, a self-evaluation system has been developed to empower farmers to assess their compliance with animal welfare standards. This system uses specific checklists and manuals that allow farmers to independently evaluate their practices, fostering a sense of responsibility and motivation to improve animal welfare [13].

Some authors have developed a new practical welfare assessment protocol for dairy farms, providing a quicker alternative to the extensive Welfare Quality protocol [14]. This new Welfare Monitor can be completed in 1.5 h and has been well-received by farmers, leading to substantial improvements in welfare conditions and financial outcomes [14].

A protocol for assessing the welfare of camels on farms has been developed by Padalino et al. [15]. These checklists not only help to ensure compliance with welfare standards but also help to improve the health and productivity of farm animals [15].

However, there are still some shortcomings for ostriches: there is no specific legislation on the welfare of this species in breeding, and no official checklists and/or protocols have been developed, highlighting the urgent need for adequate tools to guarantee the welfare of this new farmed species. This last aspect is crucial, as having an official protocol would allow a complete and objective assessment of the conditions on the farm, through the attribution of scores, as occurs in the main species of zootechnical interest. For the latter, official checklists have been developed over the years that include both indirect and direct indicators: the former relate to environmental conditions and therefore to all aspects that indirectly influence welfare, while the latter refer directly to the animal—in terms of its behavior, health, and physical condition—and are therefore defined as animal-based measures (ABMs).

This gap has prompted the need for a tailored welfare assessment protocol specifically for ostriches, which takes into account both their unique physiological and behavioral characteristics and the specific conditions of their farming environments.

This study aims to develop a feasible and time efficient protocol for evaluating the welfare of ostriches raised in intensive and semi-intensive systems, designed to offer a standardized and easy-friendly tool for assessing individual farms.

It could also help to develop quality certifications for animal-welfare-friendly products, identify welfare risk factors, and support new legislative proposals on ostrich welfare.

## 2. Materials and Methods

### 2.1. Selecting Indicators

The welfare indicators for ostriches were selected from a previous systematic review [16] that focused on assessing various aspects of ostrich welfare, including behavior, management, physiology, and pathology. Due to the limited existing literature specifically addressing ostrich welfare, this study adapted established welfare frameworks to suit this species. In particular, it incorporated criteria and principles from the Five Domains Model [17] and the Animal Welfare Indicators Project (AWIN), which had to cover species not considered in the Welfare Quality^®^ protocol [18], aligning with the principles outlined in Directive 98/58/EC [19].

The Five Domains Model was used to evaluate ostrich welfare across five key areas: nutrition, environment, health, behavior, and mental state. This model was adapted to address species-specific welfare challenges by focusing on relevant stressors and well-being indicators that are crucial for ostriches in farming systems.

The AWIN protocol, commonly used for various farmed species, was tailored by adapting its behavioral, physiological, and environmental welfare indicators to the unique needs of ostriches. This included modifications to better assess the specific behavioral and environmental factors that impact ostrich welfare in intensive and semi-intensive systems.

The Welfare Quality^®^ Protocol, a widely recognized framework for farm animal welfare assessment, was utilized as a guiding structure. This protocol, which covers aspects such as physical condition, emotional state, and freedom from distress, was modified to incorporate the particular needs and responses of ostriches in intensive and semi-intensive farming systems.

The selected welfare indicators were integrated into a comprehensive assessment protocol, ensuring that the evaluation of ostriches’ welfare was both scientifically grounded and practical for use in farming environments.

The selection process was further refined by an animal welfare expert (an author, a diplomate in ECAWBM-sub. AWEL) and by consulting relevant literature [16]. To ensure practical applicability, the inclusion criteria prioritized indicators that were readily measurable and objective [20,21], feasible for implementation in commercial settings, and did not require invasive procedures or pose risks to animal or human safety, as supported by the literature for other species [15,22,23,24]. This approach excluded indicators requiring extensive laboratory analyses (e.g., metabolic profiling) and those necessitating close physical contact, due to the potential for ostrich aggression [25,26], as well as biosecurity and drug-related criteria.

### 2.2. Composing the Protocol

The protocol developed for assessing ostrich welfare (Ostrich Welfare Assessment Protocol—OWAP) integrates a combination of direct and indirect parameters through a comprehensive checklist. This multi-faceted approach allows for a thorough evaluation of welfare by covering three main categories: animal-based, resource-based, and management-based measures.

Direct parameters, which are those that can be observed or measured directly from the animals themselves, provide immediate insight into the welfare status of the ostriches. These include factors such as their behaviour, physical condition, and health status. In this study, 14 animal-based measures (ABMs) were included, focusing on observable aspects of ostrich welfare.

Indirect parameters assess the conditions surrounding the animals, rather than their immediate physical or behavioral states. These are factors that may influence the welfare of the ostriches but are not directly observable from the animals themselves. In this protocol, 12 resource-based measures (RBMs) and 15 management-based measures (MBMs) were included. These parameters measure aspects such as the environmental conditions and the management practices under which the ostriches are kept, which indirectly affect their welfare. RBMs examine the quality and adequacy of resources provided to the ostriches, such as housing conditions, space allowance, feed quality, and water access. MBMs focus on the decisions made by farm managers, such as stocking density, feeding practices, health monitoring, and staff training. These parameters influence the overall welfare environment but are indirectly related to the animals’ immediate condition.

The checklist draws upon existing welfare assessment protocols for production animals, particularly broiler chickens, with modifications made to address the species-specific physiological and ethological needs of ostriches. The welfare indicators for ostriches were selected from a previous systematic review [16], which focused on the assessment of different aspects of ostrich welfare, including behavior, management, physiology, and pathology. For example, these indicators were selected based on aspects such as the ostrich’s monogastric herbivorous digestive system requiring a fiber-rich diet, the need for large spaces to prevent abnormal behaviors, the importance of a varied substrate that mimics the natural environment, and the stress factors relevant to ostriches in farming systems.

### 2.3. Scoring System of the Indicators

To ensure a systematic and objective assessment, a scoring system was developed for the welfare indicators. Each parameter, whether direct or indirect, was assigned a score based on its observed or measured state. The scoring system typically followed a graded scale, where the welfare status of the animal or condition is rated, as reported in Table 1.

These scores are then aggregated to provide an overall welfare assessment. A threshold has been set to determine whether the welfare of the ostriches is considered acceptable, suboptimal, or unacceptable, based on the total scores across the criteria (95 total score, 64 for indirect measures and 31 for direct measures). A score <32 is considered not acceptable, between 32–63 suboptimal, and >63 optimal. This allows for a comprehensive understanding of the welfare status and facilitates comparisons across different farming systems.

## 3. Results

### 3.1. Ostriches Welfare Assessment

Table 1 provides a comprehensive overview of the ostrich welfare assessment, categorized into indirect (resource and management-based) and direct (animal-based) measures. It lists the four welfare principles (Good feeding, Good Housing, Good Health, Good Management) and related criteria and scores.

#### 3.1.1. Indirect Measures

The OWAP relating to the indirect measures takes into account the following parameters:

(i) Good feeding (appropriate nutrition): daily feeding frequency, feed quality, feed cleanliness, access to food; absence of prolonged thirst: daily water supply frequency, water quality, access to water, frequency of inspection of watering systems;
(ii) Good housing: Suitable environment (outdoor environment, substrate, shelter, resting area, temperature and humidity, enclosures, access doors to indoor areas) and Environmental enrichment: nests, mud bath tub or area, shrubs, pebbles;
(iii) Good management: cleaning of outdoor areas, cleaning of cemented areas/equipment, daily inspections;
(iv) Good health: injured/sick animals, infirmary rooms, health planning, emergency slaughter, veterinary referral.

(i) Relating to Good Feeding, the following welfare criteria were considered:

*Appropriate Nutrition*

The feeding strategy for ostriches should prioritize constant access to food (ad libitum) to support continuous digestion and energy production. This approach ensures that the birds can eat whenever they need, rather than being restricted to specific feeding times. Feed quality is also paramount, and diets should be formulated to meet the specific needs of the birds at different life stages, such as pre-starter, starter, fattening, finishing, and maintenance phases. It is also crucial to maintain cleanliness in feeding areas by removing stale or contaminated feed before adding fresh food. Furthermore, all animals must be able to access food simultaneously, which may require a space of at least 60 cm per ostrich to prevent competition and promote even growth.

*Absence of Prolonged Thirst*

Access to clean water is equally vital. Water should be available ad libitum, particularly since ostriches in captivity often consume mainly dry feed. The water should be clean and maintained at a suitable temperature, ideally between 12–15 °C. To prevent competition and ensure all birds have access to water, it is important to provide multiple watering points. Additionally, watering systems should be inspected frequently, at least twice daily, to identify any issues and eliminate potential contamination.

(ii) The following aspects were considered to ensure Good Housing:

*Suitable Environment*

The living environment for ostriches should meet specific criteria to ensure their physical and psychological well-being. The minimum housing requirements to hold an ostrich are at least 400 square meters up to two individuals, plus an additional 15 square meters for each additional adult, to prevent overcrowding and behavioral problems. The ground should consist of a mixed substrate, including solid surfaces such as cement or concrete, but more importantly, soil, sand, and grass, to replicate their natural habitat and discourage abnormal behavior such as pica. Providing sufficient shelter to protect ostriches from the sun, wind, and predators is also essential. Indoor areas with shelters should account for at least 20% of the external space. The temperature and humidity of the environment also need to be controlled, with extreme conditions avoided through proper ventilation. Enclosures should be made from smooth materials to prevent injuries, and access doors to indoor areas should be wide and high to allow easy movement and ventilation.

*Environmental Enrichment*

Enhancing the ostriches’ living space involves providing elements that cater to their natural instincts and behaviors. Nests should be shallow depressions in the ground or sand and kept dry, with shrubs and straw to protect eggs from moisture. Mud bath areas should also be provided to encourage natural cleaning behaviors. The presence of shrubs supports their exploratory behavior, and pebbles should be available to assist with digestion.

(iii) Relating to Good Management, proper management practices are critical and have therefore been included. They include daily cleaning of outdoor areas to remove waste, as well as scheduled cleaning of cemented areas and furnishings to prevent waste accumulation. In addition, daily inspections are also necessary to monitor the health of the birds and to identify any dead or escaped animals.
(iv) Criteria relating to Good Health include absence of injuries and of disease, as well as the presence or absence of infirmary rooms, health planning, emergency slaughter, and veterinary reference. Ostriches showing signs of injury or illness should be treated promptly, and farms should have suitable infirmary rooms to separate sick animals. Preventative healthcare, such as implementing vaccination plans, is crucial to minimize the need for medication. Farms should also have the means to carry out emergency slaughter when necessary, and it is ideal to have access to veterinary services.

#### 3.1.2. Direct Measures

Among the direct measures, the 14 ABMs were chosen. Specifically, the welfare of ostriches can be assessed through the Body Condition Score (BCS), which is a useful tool for assessing an animal’s physical state, and the Bucket Test, which measures latency time, and the amount of water consumed to evaluate thirst. It is important to observe the ostriches’ resting patterns, their use of shadows, and any stereotypic behaviors. Checking for lesions, lameness, and the condition of their feathers is also crucial. Additionally, monitoring for ectoparasites and diarrhea is vital. It is equally important to ensure that live ostriches are not plucked, as this causes stress, and that beak trimming of chicks is avoided. Furthermore, pain should be prevented in farming practices. In ostriches, specific species-related behaviors are associated with good welfare, reflecting positive affective states and overall well-being. Behaviors such as dustbathing, running, and dancing are natural activities that contribute to both the physical and mental health of ostriches [27].

## 4. Discussion

The evaluation of animal welfare on farms is a critical aspect of ensuring ethical and sustainable farming practices [10,11,12,13,14,15,16,17,18,19,20,21,22,23,24,25,26,27,28]. The protocol proposed in this study provides a comprehensive framework for assessing the welfare of farmed ostriches. It considers both management and environmental aspects alongside the physical and behavioral conditions of the animals. Ostrich welfare has become an increasingly important topic as the demand for ostrich products such as meat, eggs, feathers, and leather grows. Despite their economic potential, there is a significant gap in understanding the welfare needs of ostriches, particularly in farming environments [16]. The welfare of ostriches is influenced by various factors, including health management, feeding, reproduction, and transportation [16].

*Appropriate nutrition*

Appropriate nutrition is an important welfare criterion. Ostriches are monogastric herbivores with a digestive system adapted to digest fiber-rich plant materials. This unique adaptation allows them to utilize diets that are high in fiber, which is crucial for their health and welfare [29]. It is important to avoid ingredients like cottonseed cake, which can cause gossypol poisoning, and to ensure that salt in their diet does not exceed 0.5% [29]. A balanced diet that meets the ostriches’ needs for metabolizable energy, protein, and amino acids is essential for optimizing production and maintaining welfare [30].

To prevent leg abnormalities in ostriches, it is advisable to limit dietary protein concentrations and use higher fiber diets. This approach helps control weight gain, which is crucial for their development [1]. Additionally, providing a single life-cycle diet can be a practical solution for small flocks to meet nutrient needs and keep feed fresh. Feeding systems should also consider the ostriches’ natural feeding behavior to ensure adequate intake and prevent nutrition-related diseases [30]. Environmental factors, such as winter climatic conditions, can affect ostrich behavior and their ability to forage, which in turn impacts their water and nutritional needs. During colder months, ostriches require more energy for thermoregulation, necessitating increased feeding and water intake [31]. Therefore, it is crucial to provide adequate shelter and concentrate rations during these periods to maintain their welfare.

In general, considering the feeding behavior of ostriches in the wild and the adaptation of their digestive tract, a constant food supply (ad libitum) would be optimal to ensure continuous fermentation and digestion, and therefore energy production [32]. Moreover, ad libitum feeding, especially of green forage, helps to extend the duration of food intake, preventing it from being so brief that it leads to stereotypies such as feather picking, ground pecking, or parts of the enclosure [33]. However, this feeding method is not entirely suitable for chicks, for which, on the contrary, it would be preferable to divide their feeding into two to three meals a day to avoid problems associated with rapid growth, such as leg issues and general skeletal disorders [34]. The feeding of ostriches is a fundamental part of farm management, given its influence on both overall well-being and productive performance [35], as well as on the development and survival of chicks [36]. Different diets should be formulated depending on the production stage, containing approximately the nutrient percentages, as reported in the Table 2.

A previous study has shown that protein requirements decrease with age [37]. Recommended intake of calcium, phosphorus, and fiber are given in Table 3 [32]. 

During the reproductive phase, due to the increased need for both energy and calcium and phosphorus for egg production, the diets for females should contain at least 16 g of calcium per kg of feed and a minimum of 8.5 MJ of metabolizable energy (ME) [33]. For male breeders, it is necessary to adjust the diet to avoid fertility issues [34]. Specifically, during the peak production period, the daily protein requirement is around 180 g, which decreases (to 120 g/day) by the end of the breeding season [38]. In general, the protein content in diets for ostriches should not exceed 20%, as excessive amounts could negatively affect egg production and hatching [35].

Cleaning the feed and the trough is necessary to prevent the proliferation of molds and bacteria, which can be harmful to the animal if ingested. Additionally, they may attract rodents and wild birds that can transmit various diseases [39].

All animals should have simultaneous access to food to avoid problems such as increased juvenile mortality, inadequate growth, and poor weight gain. These issues may arise due to competitions, where dominant birds consume all the food, leaving shyer birds without access. This leads to significant heterogeneity in size and weight. Therefore, the space provided should be at least 60 cm per ostrich, considering that the body width is much greater than that of the neck and head [39].

*Absence of Prolonged Thirst*

Access to fresh, clean water is vital for ostriches, as their water economy is similar to that of other large savannah and desert animals. The ratio of water intake to dry matter intake remains relatively constant, emphasizing the need for consistent water availability to prevent dehydration and welfare issues. Especially in captivity, where ostriches are primarily fed dry feeds, it is crucial to provide water ad libitum and ensure it is always available [33]. In natural conditions, however, since they mainly consume fresh forage with high water content, ostriches are able to store a large amount of water and can tolerate dehydration up to 30% of body weight loss. Therefore, while they can go without drinking for a certain period, if water is available, they will readily consume it, especially during hot and dry periods like summer, with an adult ostrich consuming up to 18 L of water per day [40]. The main issue resulting from insufficient water intake is dehydration, which initially manifests through dense, whitish urine and later with a complete cessation of liquid excretion [34]. Furthermore, water deprivation for 24 h can reduce feed consumption by up to 45% [39]. The water provided to ostriches should not be dirty, nor should it be too hot or too cold, as this could cause refusal to drink. The temperature should be maintained at around 12–15 °C, similar to the conditions of freshwater underground water. Once again, low water quality can lead to refusal to drink, causing dehydration and metabolic diseases [39].

Considering the possible competition in an enclosure (especially if more than one male is present) and the potential negative effects of inadequate water intake, it is essential to provide multiple drinking points spaced apart, so all animals can access water simultaneously [39]. Additionally, the drinking systems should be large enough to allow simultaneous access for all individuals and maintained in suitable conditions to ensure that the water remains clean and free of excessively muddy areas [41]. It would be optimal for drinking systems to be inspected at least twice a day, to quickly identify malfunctions or breakages that could disrupt the water supply and predispose animals to dehydration. Inspections also help to prevent the environment from becoming unhealthy and ensure any feed particles that might contaminate the water are removed [39]. Moreover, especially when using a new water source, it is important to test it to determine its mineral content and microbiological contaminants. If the water comes from natural sources, more frequent monitoring is necessary due to the variable composition [33].

*Suitable Environment*

Overcrowding and confinement are the main causes of abnormal behaviors such as aggression, feather pecking, and pecking of legs and heads [42,43]. Therefore, it is essential to provide adequate space for the animals at all stages of breeding. In particular, overcrowding in chicks causes stress that results in reduced weight gain, poor feed efficiency, and increased susceptibility to infections [39]. Adult ostriches are generally kept in pairs or trios, with approximately 1672 square meters per pair (about 800 square meters per bird) and about 550 square meters per bird for trios. Colony breeding for breeders also uses spaces ranging from 12,140 to 20,234 square meters, accommodating 15 to 20 ostriches [39]. Additionally, Australian animal welfare guidelines define minimum space requirements for ostriches: 400 square meters for two animals, plus an additional 15 square meters for each extra adult [44].

Within an ostrich enclosure, the substrate should include not only a solid portion (such as cement or concrete) but also, and most importantly, soil, sand, and grass. These elements mimic the ostrich’s native environment in South Africa [44] and provide necessary environmental enrichment to prevent abnormal behaviors such as pica (massive ingestion of feces). This behavior is more common in animals raised on solid substrates without enrichments [42]. Furthermore, excessively hard substrates may predispose animals to joint pathologies such as tibiotarsal rotation (TTR), which can be triggered by numerous factors including potential traumas, genetic issues, nutritional problems, incubation-related factors, or concurrent diseases [45].

To provide shelter from both the sun and the wind, as well as protection from potential predators or aggressive companions, an adequate-sized shelter should be included within the enclosure [39]. The shelter should cover at least 20% of the surface area, allowing all birds to take refuge simultaneously and ensuring adequate movement space [44]. While this may incur additional costs, it is necessary, particularly in harsh climates or when there are aggressive specimens. Shelters should be available for all age groups, from chicks to adults [46]. Additionally, shelter is important for facilitating handling and examinations [39].

Like all areas within the enclosures, the rest area should also be maintained in suitable conditions, ensuring it is sufficiently dry and clean [39]. Excessively humid or particularly dirty areas pose a problem especially for chicks, as they are more susceptible to infections [47]. For adults, these conditions can also facilitate the entry of rodents, leading to the spread of diseases such as Salmonellosis [41].

Excessively high or low temperatures can be harmful to ostriches [44]. Therefore, to maintain good welfare conditions, it is essential to control both temperature and humidity in breeding areas and avoid extreme conditions through ventilation systems and temperate zones. This is crucial especially for chicks, who, as previously mentioned, are particularly sensitive to both high and low temperatures [47,48]. For this reason, the temperature should be maintained at 35 °C during the first week of life and then decreased by 3 °C each week. Adult ostriches are capable of maintaining their body temperature across a wide range of ambient temperatures, reaching up to 56 °C [49,50]. One study measured average peritoneal temperatures between 37.2 °C and 38.5 °C in both young and adult ostriches under winter conditions with average ambient temperatures of 2.5 °C [51]. Another study suggests that the optimal egg-laying rate occurs when the maximum daily temperature is around 20 °C [51].

Regarding humidity, to keep it at appropriate levels, ventilation systems and/or openings along the sides of the enclosures should be arranged [52]. High humidity (up to 90%) in combination with heat can cause heat stress in adult ostriches. Adequate ventilation is crucial in all stages to maintain optimal air quality and support thermoregulation and hatching [49]. In poorly ventilated structures, ammonia can accumulate, potentially exceeding 50 ppm, which causes primarily respiratory issues [39]. Additionally, it is important to protect the animals from excessively high temperatures by providing both artificial and potentially natural shade. Misting sprays can also be used to cool the birds during the hottest hours [39].

Enclosures should be constructed in a way that prevents injuries or trauma to the animals. They should feature rounded and smooth surfaces rather than rough ones. To achieve this, the sides of the enclosure could be covered with sheets or laminated panels. Additionally, enclosures made of interconnected metal wires are excellent for growing birds, as they are quite elastic and do not pose a problem when the animal bumps against them [52]. The enclosures should also be relatively high (at least 1.8 m) and should have protection at the apex to prevent ostriches from injuring themselves on any protrusions [44].

To facilitate ventilation and the passage of animals, where provided, openings to internal areas should measure at least 1.2–1.5 m in width and at least 1.8 m in height [39]. Intensive farming systems often involve high stocking densities and limited space, which can lead to stress and health issues in ostriches.

Research indicates that such environments may not adequately meet the behavioral and physiological needs of ostriches, potentially leading to welfare concerns such as increased stress and reduced reproductive performance [16,17,18,19,20,21,22,23,24,25,26,27,28,29,30,31,32,33,34,35,36,37,38,39,40,41,42,43,44,45,46,47,48,49,50,51,52,53,54]. The lack of adaptation to intensive farming conditions is highlighted as a significant factor affecting welfare, with inadequate egg production and high mortality rates being common issues [54]. Semi-intensive systems, which provide more space and opportunities for natural behaviors, are more commonly used and may offer better welfare outcomes. These systems allow for some level of free-range activity, reducing stress and improving overall health. However, they also pose challenges, such as increased exposure to environmental stressors [55]. The welfare of ostriches is heavily influenced by climatic conditions. In regions with wet and cold weather, such as Germany, ostriches tend to use open stables more frequently for protection. It is crucial to provide adequate shelter to protect them from adverse weather conditions, which can otherwise impair their well-being [56]. In northern European climates, providing both shelter and additional feed during winter is essential to meet their increased energy requirements for thermoregulation [31]. Space requirements are critical for ostrich welfare. Overcrowding can lead to stress-related behaviors, such as feather pecking, which is a common issue in ostrich farming. Ensuring adequate space and managing stocking densities can help mitigate these stressors. Factors such as temperature, air quality, and group size also play a role in maintaining a suitable environment [57].

Within an ostrich enclosure, the substrate should consist of not only a solid portion (such as cement or concrete) but also, and most importantly, soil, sand, and grass. These elements not only mimic the ostrich’s natural habitat (South Africa) [44] but also provide necessary environmental enrichment to prevent the development of abnormal behaviors such as pica (massive ingestion of feces); this behavior is indeed more pronounced in animals raised on solid substrates devoid of enrichments [42]. Furthermore, excessively hard substrates may predispose animals to joint pathologies such as tibiotarsal rotation (TTR), which can be triggered by numerous factors including potential traumas (as well as genetic, nutritional, incubation-related factors, or concurrent diseases) [45].

*Management*

The role of human interaction in ostrich welfare is also significant. Early and gentle human handling can reduce stress and improve the welfare of ostriches by making them more accustomed to human presence, which is beneficial in both intensive and semi-intensive systems. This approach can mitigate some of the negative welfare impacts associated with farming practices by reducing fear and stress responses in ostriches [26].

It is essential that that the main area is cleaned at least once a day, preferably more frequently, by removing excess feces, old food, and any other waste present. Weeds should also be uprooted from the enclosure, as they could potentially harm the animals [44]. Moreover, it is important to properly clean the wetter areas, particularly around the drinking troughs, to prevent the proliferation of mold and bacteria [39].

As with the outdoor areas, other parts of the enclosure require scheduled cleaning to avoid the accumulation of waste materials that could harm or compromise well-being. In particular, the drinking troughs should be cleaned daily to keep the water always clean and fresh. Movable parts of the enclosure and the cemented areas should be adequately cleaned and disinfected at least once a week to remove pathogens. Finally, to preserve the skin and feathers of the ostriches, the substrate should be replaced monthly or bi-monthly. However, in the case of wear or heavy rains, it would be advisable to replace it more frequently, at least partially [44].

The semi-intensive approach is often seen as a compromise between productivity and welfare, but it requires careful management to prevent issues like parasitic infections and gastric impaction, which are prevalent in these settings [58].

Like other farm animals, ostriches kept in captivity should be inspected at least once a day, to identify issues such as dead or escaped animals and to detect any hatched eggs for collection [44]. This is crucial, as eggs left too long in nests are more likely to have a higher bacterial load and may not hatch [59]. Daily inspections also help highlight any damage to the fencing that could injure or allow animals to escape, as well as identify any behavioral, health, or physiological abnormalities [44].

The importance of daily animal inspection is also emphasized in Directive 98/58/EC, which lays down minimum standards for the protection of animals kept for farming purposes. This directive stresses the need for regular inspections to ensure animal welfare. As stated in the directive, animals with injuries and/or illnesses should be treated promptly to avoid unnecessary pain and suffering, and if treatments are ineffective, a referring veterinarian should be consulted. This concept was reiterated in guidelines issued in Switzerland in 2023 [60]. Additionally, as the market expands, breeders have realized that consulting a veterinarian is beneficial for increasing productivity and reducing costs, especially through preventive health measures [46].

*Health*

Farms should have infirmary facilities to separate sick and/or injured animals when necessary. These facilities should be equipped with dry, clean bedding, and the supply of water and food should be adequate (preferably ad libitum). Furthermore, these areas must be positioned so that sick animals are physically separated from healthy ones. The presence of this quarantine area is particularly important when it is necessary to prevent the spread of bacterial or parasitic diseases in the farm [34].

As mentioned earlier, ostrich breeders should focus on preventive health measures to avoid the use of medications and the associated risks [46]. For this reason, it would be optimal for farms to develop and implement prophylactic plans that include compliance with all parameters mentioned thus far regarding their impact on the welfare and health, as well as direct measures such as vaccinations. For example, maintaining adequate nutrition serves as a form of prophylaxis, as it can prevent conditions like clostridial enteritis, although there is also a vaccination for Clostridium perfringens. Other diseases for which vaccination may be recommended include smallpox, Newcastle disease (in endemic areas), anthrax, etc., while for avian influenza, prophylaxis consists of removing sick animals and reducing contact between birds [46]. Additional preventive measures that can be applied to prevent disease transmission on farms include the “all in—all out” method, proper separation of farming environments, preventive quarantine for newly introduced animals, limitations on visitor and vehicle access, as well as attention to the entry of wild animals [41].

As in other species, in the case of particularly contagious diseases or severely compromised health conditions, emergency killing should be possible on the farm for ostriches [4]. Indeed, Regulation (EC) 1099/2009 [61] states: “The euthanasia of livestock in severe pain, in the absence of viable economic solutions to relieve suffering, is a moral duty”. For example, the method developed in South Africa for this practice involves using a captive bolt and burying the carcasses in a previously lime-filled pit, covering them with at least 2 m of soil [4].

*AMBs*

In relation to ABMs, there is currently no reference to BCS for ostriches and other poultry species, so we proposed a scoring scale that could be useful, as “A poor body condition may reflect a previous state of prolonged starvation or disease” [62]. The scale was designed with five classes, each expressing a different body condition in increasing order, as indicated in Table 4.

The “bucket test” is a method proposed as an animal-based indicator to verify the absence of prolonged thirst in a protocol for assessing the welfare of camels; it was previously used for the same purpose in horses [15]. The test involves filling a bucket with fresh water, placing it at a certain distance from the animals, and measuring:

- Latency time (in seconds): the time it takes for the animals to approach the bucket;
- Volume of water consumed (in liters): the amount of water consumed.

Clearly, latency time can be influenced by the character and curiosity of the animals. Therefore, failure to approach or actual approach does not necessarily correlate with thirst. The same variability can be observed for the volume of water consumed, as it heavily depends on both the season (it will be greater in summer) and the animals’ diet. If the diet consists mainly of dry foods, the amount of water ingested will be significantly higher than for animals that also have access to fresh, water-rich forage [15].

Assessing the presence of resting behaviors, use of shade, and stereotypies can help determine whether the farming environment is comfortable, both for poultry and other species, including ostriches. A typical resting behavior of the ostrich is sitting, which is also one of the principal behaviors recorded in farming, along with environmental exploration and standing [63]. Therefore, if the farming environment is comfortable, ostriches will naturally display this behavior. The use of shade implies that there is enough for all animals in the enclosure. If not, competition would develop among individuals, to the detriment of shyer ones, which could consequently suffer from excessive heat. Finally, stereotypies are vital indicators as they directly reflect any stress conditions the animals are exposed to. In particular, as previously mentioned, all major stereotypies in ostriches are primarily caused by excessive confinement, stress, and boredom (lack of environmental stimuli), inadequate facilities and management, as well as an unsuitable diet for the species [42,43,44,45,46,53].

In broiler chickens, weekly mortality is considered the only among ABMs, but other indicators such as the presence of injuries, lameness, and feather conditions can still be considered to assess the health conditions of the subjects and confirm any non-compliance found [64]. Such assessments can also be adapted to ostriches, which—under unsuitable housing and management conditions—may present injuries mainly due to poorly constructed enclosures, high density, unsuitable substrate, or aggression from peers. In particular, EFSA [65] has shown that high densities, resulting in increased contact between animals, can cause injury development. Lameness can also be caused by the same factors, but specifically in ostriches, it can also be observed in cases of limb problems, such as TTR, a condition very common in young animals, caused by both excessively rapid growth due to improper feeding in early stages and genetic factors [45].

Regarding feather conditions, although there is currently no official assessment for ostriches, a scoring system has been developed, where the maximum score corresponds to the ideal condition and the minimum value to the worst:

Score 1: severe damage to feathers with no or few covered areas;

Score 2: severely damaged feathers and the presence of bare patches on the back larger than 5 cm^2^ (naked up to 75%);

Score 3: skin still completely or almost completely covered with feathers, with bare patches smaller than 5 cm^2^; Score 4: complete feather cover with few worn feathers.

Another useful evaluation concerns identifying any ectoparasites, which can be detected by carefully inspecting the plumage. Specifically, among those found in poultry, there are: mites, ticks, lice, and fleas [62]. Although there is no evidence of specific ectoparasites in ostriches, they could be present if animals are kept under inadequate conditions, both in terms of pen cleanliness and insufficient prophylactic treatments applied. The presence of diarrhea may also indicate inadequate management practices, particularly inappropriate diets and/or lack of prophylactic treatments aimed at reducing and/or eliminating potential intestinal infections. Specifically, as previously mentioned, ostriches have a particular intestinal configuration that requires a diet rich in forage for proper growth and maintenance [37]. Therefore, an unbalanced diet in this species could lead to this issue.

Additionally, the presence of visible pain in animals could be associated with improper management practices and lack of adequate care. It is therefore one of the measures that can be used to assess the health conditions of ostriches.

In light of the above, the presence of a reference company veterinarian is essential for intervening in cases where the care provided on the farm is ineffective.

Live plucking of ostriches should not be carried out, as it has been widely demonstrated that it negatively affects the welfare of the animals, causing stress. Furthermore, since this practice involves extracting feathers from follicles, which may be surrounded by nerve fibers, it could also cause pain in the animals [26]. For these reasons, even Swiss guidelines recommend that this practice not be performed, either by cutting or pulling feathers.

Beak trimming is part of so-called “mutilations”, for which the ClassyFarm protocol for poultry reports the conditions [66]. Specifically, as stated in the recommendations issued in Switzerland, it would be advisable not to carry out this practice, neither in poultry nor in ostriches. Instead, efforts should be made to resolve issues of aggression, such as pecking at peers and pens by placing the animals in suitable welfare conditions with adequate environmental enrichments available.

## 5. Conclusions

Overall, the welfare of ostriches in farming environments is influenced by a combination of health management, transport practices, environmental conditions, and reproductive behaviors. Addressing these factors through research and the development of specific guidelines can help improve the welfare of ostriches and ensure sustainable farming practices. Further studies are needed to fill the existing knowledge gaps and enhance the welfare standards for ostriches globally.

However, it is important to note that the protocol lacks specific thresholds or scoring systems for each element. The interpretation of the findings and the determination of welfare status require expert judgment and consideration of the specific farm context.

Further research is needed to establish clear thresholds and validate this checklist through field application. Collaboration with ostrich scientists, stakeholders, and industry members is essential to refine this protocol and ultimately propose robust welfare standards for ostriches.

## Figures and Tables

**Table 1:** Ostrich Welfare Assessment Protocol—The criteria selected in the OWAP are categorized within the framework of the Welfare Principles and the Welfare Quality^®^ criteria.

Welfare Principles	Welfare Criteria	Scoring Criteria
**INDIRECT MEASURES**		
**Good feeding**	**Appropriate Nutrition**	
	Daily feeding frequency	1 = insufficient, ≤2 times a day; 2 = sufficient, ≥2 times a day; 3 = optimal, ad libitum.
	Feed quality	1 = insufficient, ration unbalanced; 2 = sufficient, ration balanced.
	Feed cleanliness	1 = insufficient, stale and contaminated feed; 2 = sufficient, stale feed removed; 3 = optimal, clean feeders.
	Access to food	1 = insufficient, no system to prevent competition; 2 = sufficient, system to avoid competition, space ≤ 60 cm/ostrich; 3 = optimal, simultaneous feeding, space ≥ 60 cm/ostrich.
	**Absence of Prolonged Thirst**	
	Daily water supply frequency	1 = insufficient, ≤once a day; 2 = sufficient, 1–3 times a day; 3 = optimal, ad libitum.
	Water quality	1 = insufficient, dirty, too hot/cold; 2 = sufficient, clean and fresh water.
	Access to water	1 = insufficient, no system to prevent competition; 2 = sufficient, system to avoid competition, space ≥ 60 cm for ostrich or automatic system.
	Frequency of inspection of watering systems	1 = insufficient, <once a day; 2 = sufficient, once a day; 3 = optimal, ≥2 times a day.
**Good Housing**	**Suitable Environment**	
	Outdoor environments	0 = not present; 1 = insufficient, environments ≤ 400 sqm/individual + 15 sqm each additional adult; 2 = sufficient: ≥400 sqm/individuals + 15 sqm each additional adult; 3 = optimal: superior spaces.
	Substrate	0 = not present; 1 = insufficient, concrete; 2 = sufficient, soil/sand/grass.
	Shelter	1 = insufficient, no shelters; 2 = sufficient, outdoor shelter; 3 = optimal, indoor shelter for at least 20% of the external area.
	Rest area	0 = not present; 1 = insufficient: resting area not sufficiently drained; 2 = sufficient: resting area dry and clean.
	Temperature (T) and humidity (H)	0 = not present; 1 = insufficient: no temperature and humidity control; 2 = sufficient: it is possible to avoid temperature and humidity excessively hot (T > 35°; relative H > 90%) and/or cold (T < 2.5°)/systems ventilation.
	Enclosures	0 = not present; 1 = insufficient: possibility of injuries to animals; 2 = sufficient: fences in good condition without edges and/or protrusions that could injure animals.
	Access doors to indoor areas	0 = not present; 1 = insufficient: 1.8 m height (or animals don’t slow down when they enter) × <1.2 m width; 2 = sufficient: <1.8 m h × 1.2–1.5 m width.
	**Environmental Enrichment**	
	Nests	0 = not present; 1 = insufficient: they are not planned nest boxes rather, there are cassettes (which reduce the space) and/or are not sufficiently isolated; 2 = sufficient: the nests are a shallow depression in the ground (or sand); 3 = optimal: nests as above + bushes and/or straw around.
	Mud bath tub or area	0 = not present; 1 = insufficient; 2 = sufficient= water ponds, pools and mud pools of 2.3 square meters by 15.24 cm deep.
	Shrubs	0 = not present; 1 = inadequate: poor presence of shrubs; 2 = sufficient: number of shrubs so that animals can use them at the same time.
	Pebbles	0 = not present; 1 = inadequate: poor presence of pebbles; 2 = sufficient: presence of sufficient presence of pebbles for the number of animals.
**Good management**	**Management**	
	Cleaning of outdoor areas	0 = not present; 1 = insufficient: cleanliness not scheduled daily; 2 = sufficient: removal of waste material daily; 3 = optimal: cleaning several times a day.
	Cleaning of cemented areas/furnishings	0 = not present; 1 = insufficient: cleanliness not programmed; 2 = sufficient: cleanliness and scheduled disinfection.
	Daily inspections	1 = insufficient: <1 once a day; 2 = sufficient: at least 1 once per day.
	**Health**	
**Good Health**	Injured/sick	0 = not present; 1 = insufficient: not promptly treated; 2 = sufficient: immediately treated, possibly vet.
	Infirmary rooms	1 = insufficient: local absentees to separate animals if sick or injured; 2 = sufficient: local presence to separate animals if sick or injured with adequate dry and clean bedding.
	Health planning	1 = insufficient: not planned prophylaxis; 2 = sufficient: scheduled prophylaxis treatments.
	Emergency slaughter	1 = insufficient: not possible carry out felling emergency in the farm in case of necessity; 2 = sufficient: it is possible carry out felling emergency in the farm in case of necessity.
	Veterinary referral	1 = absent; 2 = occasionally present; 3 = present.
**DIRECT MEASURES**		
	**Body Condition Score (BCS)**	1 = Emaciated (BCS1) or Underconditioned (BCS2); 2 = Fat (BCS4) or Extremely Fat (BCS5); 3 = Ideal (BCS3)
	**Bucket Test**	
	Latency Time (LT/ sec.)	1 = insufficient, LT < 30 s; 2 = sufficient, LT > 30 s.
	Quantity of water drinking [QWD/l (liters)]	1 = insufficient, very QWD (>2 l); 2 = sufficient, low QWD (1–2 l).
	**Attitudes of rest**	1 = insufficient, absent; 2 = sufficient, present.
	**Using Shadow**	1 = insufficient, absent; 2 = sufficient, present.
	**Stereotypies**	1 =insufficient, present; 2 = sufficient, absent.
	**Lesions**	1 = insufficient, absent; 2 = sufficient, present.
	**Lameness**	1 = insufficient, absent; 2 = sufficient, present.
	**Feather condition**	1 = serious damage; 2 = strongly damaged and patchy; 3 = mostly covered; 4 = full coverage.
	**Ectoparasites**	1 = insufficient, present; 2 = sufficient, absent.
	**Diarrhea**	1 = insufficient, present; 2 = sufficient, absent.
	**Obvious pain**	1 = insufficient, present; 2 = sufficient, absent.
	**Plucking live ostriches**	1 = insufficient: regularly plucking of live birds; 2 = sufficient: not carried out plucking of live birds.
	**Beak trimming of ostrich chicks**	1 = insufficient (if carried out); 2 = sufficient (if not carried out).

**Table 2:** Main nutrient depending on the ostriches’ production stage [35,37].

Production Stage	Main Nutrient
Pre-starter (0–6 weeks)	High-quality fiber
Starter (6–11 weeks)	Up to 20% roughage
Fattening (11–22 weeks)	40% cereals and 16% protein
Finishing (22–37 weeks)	25% cereals, 14% protein, and up to 70% roughage
Maintenance (≥37 weeks)	10–12% protein and the remainder roughage
Slaughter	Mainly roughage (90%)

**Table 3:** Nutrient and fiber requirements at different stages of production stage.

Production Stage	Calcium Requirement (%)	Phosphorus Requirement (%)	Fiber Level (%)
Pre-starter (0–2 months)	0.8–1.8	0.8–1.8	>10
Starter (2–5 months)	0.8–1.8	0.8–1.8	>13.5
Fattening (5–7 months)	0.5–0.6	0.5–0.6	>17.5

**Table 4:** Ostriches Body Condition Scoring.

Body Condition Scoring (BCS)	Description	
**BCS 1:** **Emaciated**	Extremely thin animal with prominent bones, a sharp breastbone, and minimal muscle or fat cover.	** 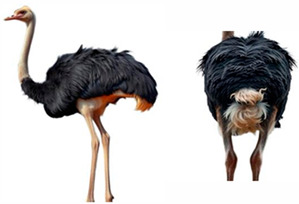 **
**BCS 2:** **Underconditioned**	Animal showing low body condition with limited muscle and fat, appearing thin and lacking body mass.	** 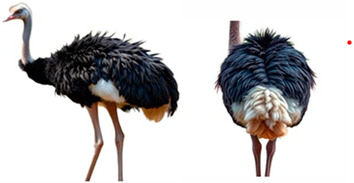 **
**BCS 3:** **Ideal**	Animal in optimal condition, with well-balanced muscle and fat, no visible bones or excess fat.	** 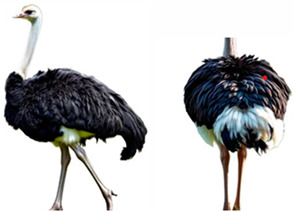 **
**BCS 4:** **Fat**	Animal with excessive fat, giving a rounded appearance.	** 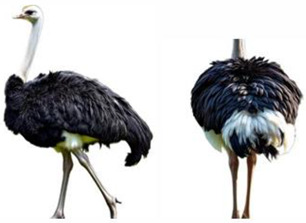 **
**BCS 5:** **Extremely Fat**	Obese animal with bone structure completely obscured by large amounts of fat and bone structure not palpable.	** 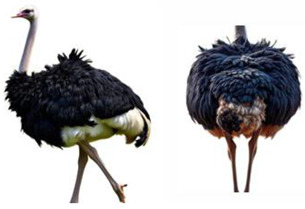 **

## Data Availability

For other information contact the corresponding author (mi-chela.pugliese@unime.it).
